# Salvage Metastasectomy After Adoptive Cell Transfer for Melanoma: Long-Term Updates

**DOI:** 10.1245/s10434-026-19354-1

**Published:** 2026-03-21

**Authors:** Aaron J. Dinerman, Alexandra M. Gustafson, Kyle J. Hitscherich, Donald E. White, Sri Krishna, David S. Schrump, Chuong D. Hoang, Jonathan M. Hernandez, Jared J. Gartner, Paul F. Robbins, Mei Li Kwong, Stephanie L. Goff, James C. Yang, Steven A. Rosenberg, Nicholas D. Klemen

**Affiliations:** 1 Surgery Branch, National Cancer Institute, National Institutes of Health, Bethesda, MD USA; 2 Thoracic Surgery Branch, National Cancer Institute, National Institutes of Health, Bethesda, MD USA; 3 Surgical Oncology Program, National Cancer Institute, National Institutes of Health, Bethesda, MD USA

## Abstract

**Background:**

Adoptive cell transfer (ACT) can mediate durable regression in patients with metastatic melanoma, and some patients with initial response may progress, requiring salvage therapy.

**Methods:**

We report updated follow-up on a retrospective review of patients with metastatic melanoma receiving ACT at a single institution from 2000 to 2022. We identified patients with objective response (RECIST 1.0) or stable disease for at least 6 months (SD6) before experiencing oligoprogression in up to three extracranial sites managed with surgery. The primary outcomes were progression-free survival (PFS) and overall survival (OS) after surgery. We also performed an exploratory analysis for genomic mechanisms of tumor escape.

**Results:**

In total, 138 patients with metastatic melanoma treated with ACT had an objective response or SD6 before experiencing disease progression. Of these, 30 (21%) had extracranial oligoprogression managed with metastasectomy. With a median follow-up of 132 months (range 5–248), median OS was not reached and median PFS was 10.5 months. Ten-year OS was 54.7% and plateaued after 6 years. Four patients with pre- and post-ACT tumor sequencing demonstrated significant mutational evolution, with one patient experiencing loss of two major histocompatibility complex class I-restricted neoantigens recognized by T cells in the original infusion product.

**Conclusions:**

Of patients experiencing oligoprogression after initial response to ACT (complete response, partial response, or SD6), 27% derived durable disease control after surgical management, confirming a potentially viable salvage option for “local” failures following ACT that may not portend systemic failure. Additional factors related to ACT response and oligoprogression that may guide patient selection for salvage metastasectomy remain undefined.

**Supplementary Information:**

The online version contains supplementary material available at 10.1245/s10434-026-19354-1.

Immune checkpoint blockade (ICB) using antibodies against programmed cell death protein-1 (PD-1), programmed cell death ligand-1 (PD-L1), and/or cytotoxic T-lymphocyte-associated protein (CTLA)-4 is established as the mainstay of therapy for metastatic melanoma.^1,2^ ICB also has a growing role in the neoadjuvant and adjuvant settings, highlighting the growing role of immunotherapy in complementing surgical resection.^3,4^ Adoptive cell transfer (ACT) of tumor-infiltrating lymphocytes (TIL) is another immunotherapeutic strategy that was approved by the US Food and Drug Administration (FDA) in 2024 after several decades of investigation.^5–7^

Both ICB and ACT are unique among systemic treatments for metastatic solid tumors in that they are capable of mediating complete responses (CRs) that are frequently durable—indicative of potential cure or at least long-term systemic disease control. An increasingly common clinical scenario in patients being treated with these therapies is that of oligoprogression, loosely defined as growth of “escape” lesions in patients who otherwise appear to be having a response following treatment with ICB or ACT.

It stands to reason that if patients who have a CR after ICB or ACT are frequently cured, then perhaps some patients with a good response to immunotherapy outside of up to three escape lesions also have systemic disease control but with isolated local failure(s). If that is the case, some patients with escape lesions may achieve prolonged disease-free survival after local therapy. Alternatively, if escape lesions are simply the first harbinger of systemic failure, then local treatment of escape lesions will, with sufficient follow-up, inevitably be followed by progression of systemic disease.

Multiple retrospective cohort studies have described outcomes in patients with metastatic melanoma undergoing treatment of escape lesions after ICB.^8,9^ Our group previously reported on local therapy for escape lesions after ACT.^10^ However, these studies were all limited by relatively short median follow-ups, ranging from 23 to 62 months.^8–10^ However, patients with metastatic melanoma who are responding to immunotherapy tend to have a long disease course, and recurrences only begin to plateau after approximately 2–3 years.^7,11^

Given the extended disease course of many patients with metastatic melanoma who respond to immunotherapy, long-term follow-up is critical to understand whether resection of escape lesions eliminated the last site(s) of immune-resistant disease or if it simply extirpated the first visible site of recurrence. This is important information because it may alter the choice of next salvage therapy (i.e. a different systemic regimen vs. surgery) and influence the risk/benefit calculation for surgery if curative resection is theoretically possible.

To address this knowledge gap, and to better understand the natural history of oligoprogressive melanoma after a response to ACT, we obtained additional prospectively recorded follow-up on a cohort of patients previously reported at this institution^10^ and included several additional patients who were treated after our prior study while meeting the original criteria for inclusion. This updated study now reports a median follow-up of 134 months and additionally afforded the opportunity to explore insights from whole-exome sequencing (WES) obtained from pre- and post-ACT specimens in several patients.

## Methods

### Patient Selection Criteria

We reviewed prospective data from patients with metastatic melanoma who were treated under clinical trials of ACT at a single institution from 2000 to 2022. The specific protocols we included were previously described^10^ and generally involved administration of lymphodepleting chemotherapy, cell transfer, and exogenous interleukin (IL-2). Next, we identified patients who had a prospectively recorded objective response (CR or partial response [PR]) by Response Evaluation Criteria in Solid Tumors (RECIST) 1.0 or who had stable disease for at least 6 months (SD6) before developing progressive disease (PD).

From this group, we then identified patients who had surgery after the date of PD to resect up to three sites, with pathologic confirmation that the resected specimen had viable melanoma. Notably, this analysis excludes patients who underwent resection of stable but non-progressing disease and those who had surgery for progression in the central nervous system (CNS) because brain metastases are managed uniquely and local therapy in the CNS is already supported by level I data.^8^ We also excluded patients who underwent surgical intervention for the sole purpose of research procurement, harvest for TIL, or symptomatic palliation.

The primary study outcomes were progression-free survival (PFS) and overall survival (OS) after surgery, with an exploratory analysis for clinicopathologic factors associated with PFS and OS. Multi-staged operations were included if they were planned and recorded as a single procedure with PFS and OS being recorded starting from the date of the final operation.

### Clinical Protocols

Patients included in the final analysis were enrolled in one of 12 clinical protocols approved by the institutional review board and involving lymphodepleting chemotherapy followed by ACT of autologous TIL or autologous T-cell receptor (TCR)-engineered peripheral blood lymphocytes (TCR-T cells). Patients had measurable disease and an Eastern Cooperative Oncology Group score of 0 or 1. The details of production and administration of these cell products in addition to clinical trial results are previously published.^4,9–11^

Briefly, TILs harvested from metastatic lesions were cultured in high-dose IL-2, expanded and administered to patients under one of 12 protocols. TCR-T products were generated by retroviral transduction of autologous peripheral blood lymphocytes obtained from an apheresis with one of four A*02:01-restricted TCRs targeting melanoma antigens (MAGE-A3, MART-1, gp-100, or NY-ESO-1).

All patients received a non-myeloablative, lymphodepleting chemotherapy regimen consisting of cyclophosphamide (60 mg/kg/day) for 2 days and fludarabine (25 mg/m^2^/day) for 5 days. Patients enrolled in protocols receiving additional total body irradiation (2 or 12 Gy fractionated over 3 days) were combined with CD34+ stem cell pre-harvest and re-infusion. After completion of the preconditioning regimen, patients then received their cell infusion with high-dose IL-2 dosing (720,000 IU/kg) every 8 h as tolerated.

### Surveillance

Following ACT, patients underwent prospectively scheduled surveillance imaging, including computed tomography of the chest, abdomen, and pelvis immediately before and within 4–6 weeks of receipt of ACT. Patients with liver metastases usually underwent magnetic resonance imaging, and additional imaging modalities were utilized per principal investigator discretion. Post-treatment imaging was conducted every 2 months for the first year with de-escalation to 3- to 6-month intervals in the next 4 years. Brain imaging was required before protocol enrollment and every 4–6 months after ACT with magnetic resonance imaging.

Post-metastasectomy imaging evaluations were initially conducted every 3–4 months in the first year and de-escalated to semiannual intervals in the next 4 years. Brain imaging was conducted in conjunction with these imaging evaluations but often at every other visit unless otherwise indicated by prior CNS disease status. Patients with progressive disease after their salvage metastasectomy were discharged unless offered an additional surgical resection and/or alternative investigational therapies.

### Pattern of Failure

Our original publication found a retrospective association between the pattern of ACT treatment failure and clinical outcomes after resection of escape lesions. Briefly, “pre-existing” escape lesions were evident on imaging before the date of ACT, whereas “new” escape lesions first appeared after ACT. These designations are preserved for the 26 original patients, whereas—for the four new patients—they were prospectively recorded in the medical record.

### Extent of Resection

The goal of surgery for patients in this study was to remove all *progressing* disease. Some patients who had surgery for escape lesions also had non-progressing residual disease (NPRD) that was stable or still responding to ACT, and these NPRD sites were not necessarily removed. If metastasectomy resulted in clearance of all remaining disease, the patient was rendered with no evidence of disease (NED). Patients resected to NPRD had stable or responding metastases that were still evident on scans.^9^

### Statistical Analysis

PFS and OS were the primary outcomes and measured from the date of metastasectomy utilizing the Kaplan–Meier method. Statistical analysis for association between outcomes in patients depending on the timing of metastasis (pre-existing vs. new site of disease) or extent of resection (NED vs. NPRD) was analyzed for correlation based on the log-rank method.

### Whole Exome Sequencing

In an exploratory analysis, we compared WES data on pre- and post-ACT treatment specimens. These patients had been previously studied as part of alternative research endpoints and/or screened for potential future treatment with additional ACT. WES of patients’ tumor and normal peripheral blood cells were used to identify somatic mutations.^4^ RNA-sequencing was performed for gene expression and transcriptome analysis. These differences were reconciled with known reactivities in the context of previous experiments conducted for T-cell recognition of neoantigens (discussed next).

### Neoantigen Screening

Three patient samples underwent retrospective research screening with tandem minigenes (TMG) and corresponding peptide pools. TMGs and peptide pools were synthesized as previously described.^12^ Autologous patient-derived immature dendritic cells or CD40L-activated B-cells were cultured^12^ and these antigen-presenting cells were then transfected with TMGs and rested overnight or pulsed with peptide pools for co-culture with TIL cultures grown from resected metastatic lesions. After co-culture for 18–24 h, experimental readout was conducted for T-cell activation with flow cytometry for 4-1BB (CD137) upregulation and ELISpot for interferon-y release. Reactive cultures were then deconvoluted with additional co-cultures with individual peptide elements of corresponding reactive peptide pools or TMGs. Human leukocyte antigen (HLA) restriction was conducted as previously described using COS7 cells transfected with patient-specific class I HLA plasmids.

In some instances where TIL fragments were grown from the metastasectomy specimen, these T-cell cultures were also tested for neoantigen reactivity.

## Results

**Fig. 1:**
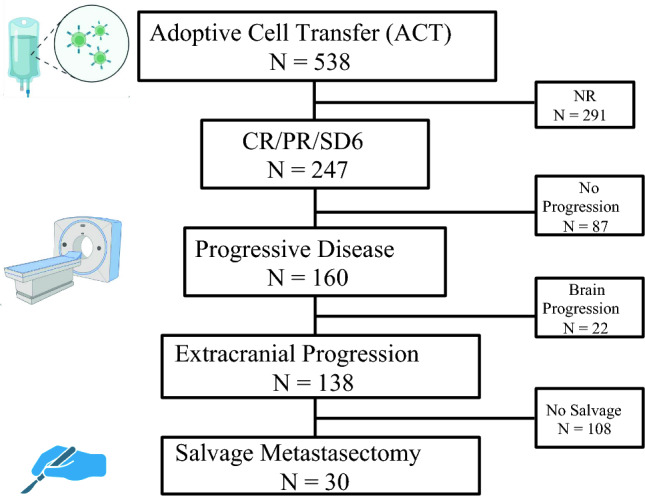
Flow diagram for cohort patient selection. CR, complete response; NR, PR, partial response; SD6, stable disease for at least 6 months.

Our original cohort described 115 patients who had a CR, PR, or SD6 after ACT before developing extracranial PD, 26 (23%) of whom underwent local therapy for up to three escape lesions.^10^ This updated cohort now includes a total of 138 patients with a CR, PR, or SD6 before PD, and 30 (22%) had surgery to control escape lesions. These 30 highly selected patients represent the focus of this updated cohort (Fig. 1).

In total, 108 patients with a CR/PR/SD6 who progressed after ACT did not undergo salvage metastasectomy. Surgery was not performed in these patients for a variety of reasons, including technical considerations, clinician judgment, and patient preference, but anecdotally the most common reason was that progression was multifocal.

Among the 30-patient cohort who did have salvage surgery, most had M1c melanoma at the time of ACT (*n* = 23 [76.7%]) and had received a variety of prior systemic therapies before index cell therapy (Table 1). It is noteworthy that the most common systemic therapy before ACT was IL-2 (*n* = 19 [63.3%]). The number of patients who received CTLA-4 or PD-1/PD-L1 blockade before ACT was 10% and 3%, respectively. ACT itself involved transfer of TIL in most patients (*n* = 24/30 [80%]), whereas a small proportion received TCR-T (Table 1, *n* = 6/30 [20%]).

**Table 1:**
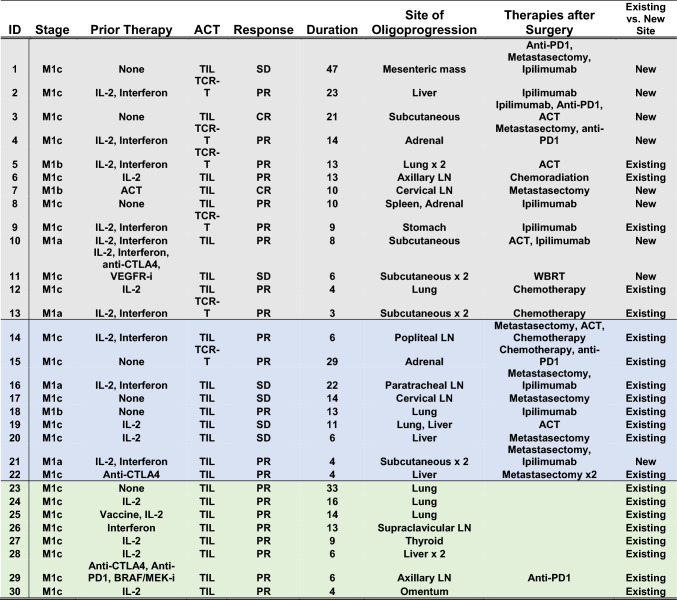
Clinical characteristics of all patients included in metastasectomy cohort

Gray shading indicates patients with progression and expiration after metastasectomy, blue shading indicates patients with progression without expiration after metastasectomy (alive with disease), and green shading indicates patients without progression after metastasectomy (progression-free alive). ACT Adoptive cell transfer, CR Complete response, CTLA Cytotoxic T-lymphocyte-associated protein, IL Interleukin, LN Lymph node, PD1 Programmed cell death protein-1, PR Partial response, SD Stable disease, TCR-T T-cell receptor-engineered peripheral blood lymphocytes, TIL Tumor-infiltrating lymphocytes, VEGFR-i Vascular endothelial growth factor receptor-1, WBRT Whole-brain radiation therapy

**Fig. 2:**
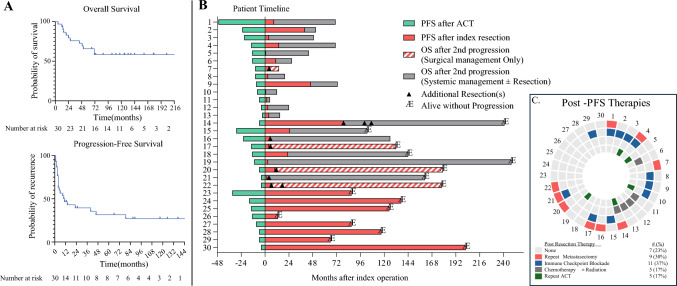
**A** Kaplan–Meier curves showing overall survival (OS) and progression-free survival (PFS) for the cohort of 30 patients who had surgery for oligoprogression after an initial response or stable disease. **B** Swimmer’s plot showing the duration of response or stable disease following adoptive cell transfer (ACT) in green, PFS following the initial surgery in red, OS following a second progression in grey (systemic management ± resection) and red stripe (surgical management only). Arrows indicate ongoing survival. Triangle icons indicate additional resections. **C** Chart showing local or systemic therapies administered after post-surgical progression

For the updated cohort of 30 patients, the median follow-up was 132 months (range 5–248), median PFS was 10.5 months (Fig. 2), and 5 year OS was 63.3%. Seventeen (57%) patients were alive at last follow-up, and eight patients (27%) had ongoing PFS, indicating durable disease control. These eight patients remained without evidence of disease at median follow-up of 132 months. Ten-year OS was 57%, 10 year PFS was 27%, and both reached a plateau after 6 years.

Six (20%) of 30 patients had ACT with TCR-T cells rather than TIL before undergoing surgery for escape lesions (Table 1). At last follow-up, five of those patients had developed PD and died, but one individual who received NY-ESO-1 targeted TCR-T cells has ongoing survival despite progressing again 2 years after metastasectomy. By contrast, 57% of patients with TIL were alive at last follow-up (*p* = 0.06).

For the 22 patients who progressed again after surgery, subsequent therapies were as follows: 36% had a second surgical resection, 32% had systemic therapy with ICB, 18% had another ACT, and 14% had chemotherapy and/or radiation (14%) (Fig. 2C). Following a second surgical resection, 22% of patients had long-term PFS and required no further systemic treatments. Of the 22 patients who progressed again after their surgery, most (*n* = 16 [73%]) progressed within 1 year after surgery.

**Fig. 3:**
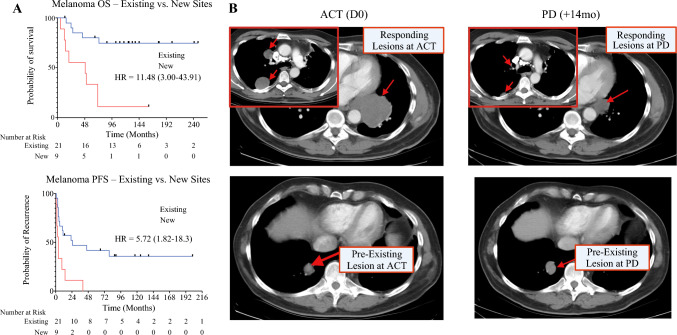
**A** Kaplan–Meier curves showing overall survival (OS) and progression-free survival (PFS) for patients with progression in existing vs. new sites. **B** Representative imaging of a patient with an initial response who experienced oligoprogression in a right pulmonary lesion at 14 months. Of note, a para-aortic lesion in the left chest continued to respond at 14 months and was not resected, representing a site of non-progressive residual disease (NPRD). ACT, adoptive cell transfer; D0, day 0; mo, months; PD, progressive disease

Re-analysis of OS and PFS related to pattern of initial treatment failure showed that resection of “pre-existing” escape lesions continued to be associated with favorable outcomes (Fig. 3A; hazard ratio 11.48 and 5.72, respectively; *p* = 0.0004 and 0.0028). All patients with durable PFS after surgery had a pre-existing escape lesion (*n* = 8/8 [100%]). Among the 17 patients who were alive at last follow-up, 16 (94%) had resection of a pre-existing escape lesion. All four of the additional patients added to this cohort since our initial publication had escape lesions that were prospectively determined to be pre-existing; two of the four have ongoing PFS, and all four were alive at last follow-up.

The extent of resection was to NED in 20 (67%) of patients and to NPRD in 10 (33%). The median PFS of patients resected to NED and NPRD was 14 and 9.5 months, respectively; median OS was not reached at 156 months (Supplementary Fig. 1). Of the 10 patients resected to NPRD, two (20%) experienced durable PFS and five (50%) were alive at last follow-up.

**Fig. 4:**
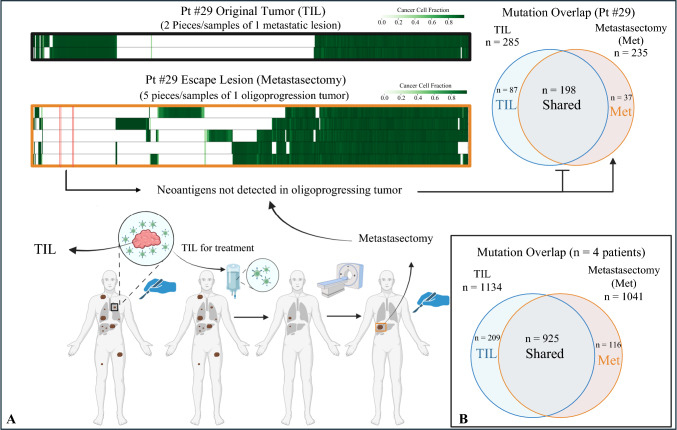
**A** Bars show non-synonymous mutations from whole-exome sequencing (WES) of patient (Pt) #29 original tumor and escape lesion with loss of two CD8 neoantigen mutations in escape lesion. Representative schema illustration and Venn diagram of shared vs. unique mutations between original tumor and escape lesion. **B** Venn diagram of mutational overlap (shared vs. unique mutations) in four patients with pre- and post-treatment sequencing. Met, metastasectomy; TIL, tumor-infiltrating lymphocytes

In four patients, WES was performed from both pre- and post-ACT specimens as well as experimental validation of TIL reactivity against unique neoantigens. First, we evaluated for loss of heterozygosity at chromosome 6 or for the presence of mutations in HLA or beta 2 microglobulin, which are well described escape mechanisms that lead to failure of antigen presentation.^13–16^ We did not identify any of these mechanisms in escape lesions. In one patient, we did identify that the escape lesion exhibited loss of two unique HLA class I-restricted (CD8) neoantigens that were recognized by CD8 T-cells in the original TIL infusion product (Fig. 4A). Despite the progressing lesion losing these two CD8 neoantigens (targeting ATL2 and GATA6), TIL cultured ex vivo from the escape lesion demonstrated novel reactivity to a neoantigen that was not detected in the original (pre-ACT) tumor specimen (LAMC1).

In two other patients, the escape lesions were confirmed to have retained expression of neoantigens that were present in the pre-ACT specimens, although in one instance the expression was sub-clonal. Aside from this, no clear mechanisms of tumor escape were identified. Overall, metastasectomy of progressing lesions demonstrated a variable degree of sequencing discordance with their original TIL harvest lesion, introducing the possibility of antigenic drift as a mechanism of evasion for these patients (Fig. 4B).

## Discussion

With the recent FDA approval of ACT of TIL for patients with metastatic melanoma, the armamentarium of systemic and local options for patients with this disease continues to evolve. Here we present data, albeit retrospective, that imply substantial variation in response patterns after T-cell transfer. Some patients treated with ACT experienced systemic progression in multiple sites, but several patients with an initial response (or prolonged SD) experienced oligoprogression limited to up to three escape lesions.

A key finding is that 22% of patients who had an objective response or SD6 after ACT experienced oligoprogression that was amenable to surgery. In this setting, surgical metastasectomy is one potential salvage option to be considered alongside other local therapies, systemic therapies, or other clinical trials. The finding that some highly selected patients who underwent metastasectomy had excellent long-term outcomes, including some with durable PFS, suggests surgery can be an effective salvage strategy to be weighed in the context of a multidisciplinary discussion.

It is also worth mentioning that seven patients who progressed after their initial metastasectomy were candidates for a second surgical operation (32%), and three of these have enjoyed long-term disease control after the subsequent procedures (Fig. 2, surgical management only). To summarize, 13 (43%) of the 30 patients had durable disease control, with one or two surgical procedures in the absence of further systemic therapy, confirmed with long-term follow-up data. This highlights the potential utility of local therapy as salvage treatment following ACT.

These clinical results also provide insights into the biology of metastatic melanoma, and how it can be influenced by ACT. In some anecdotal cases, patients had well over 10 metastases before ACT, responded, progressed in up to three escape lesions, and were apparently cured with surgery alone. This clinical result suggests that ACT sculpted a systemic problem (stage IV melanoma) into a local problem (up to three escape lesions) that is amenable to extirpation. A critical unanswered question is why the immune system was able to eradicate (or perhaps permanently suppress) systemic micrometastatic disease yet failed to eradicate the macroscopic escape lesion(s). These divergent responses between micro and macrometastastic disease potentially implicate the tumor microenvironment or the presence of resistant clones in the escape lesion that had not systemically disseminated.

Our initial report of 26 patients from this cohort demonstrated a positive association between resection of “pre-existing” sites of progression, as opposed to resection of new sites of progression, and clinical outcomes.^10^ This updated cohort reiterates that finding. A similar association was identified in a cohort of 52 patients treated with ICB for metastatic melanoma who had local therapy for escape lesions; in that study, the pattern of failure was determined by an independent radiologist who was blinded to the study hypothesis and design.^8^ However, that finding was not replicated in two other studies.^17,18^ Nonetheless, in this series, only one of nine patients (11.1%) with progression at a new site appeared to derive durable benefit from post-ACT metastasectomy, raising the possibility that this could represent a useful clinical factor to be weighed when considering salvage therapy for post-ACT progression.

Another clinical scenario detailed herein that is somewhat atypical is the decision to perform resection to NPRD—in other words, removing sites of progression while leaving non-progressive disease in situ. This practice has been reported by multiple groups independently in the literature, perhaps broadening eligibility criteria for patients who progress after immunotherapy.^8,9,18^ It is inherently difficult, and probably not necessary, to directly compare outcomes between patients rendered NED and those resected to NPRD. This is because patient selection differs substantially between these approaches, even if it has not been explicitly quantified. Most obviously, the threshold for offering surgery that results in NED is lower. By contrast, in our experience, resection to NPRD is only pursued when there is especially compelling evidence that systemic immune control remains active. Thus, patients resected to NED and NPRD have subtle differences that preclude direct retrospective comparisons; however, the relevant insight is not that one category outperforms the other but that both can achieve favorable outcomes when applied to appropriately selected patients.

Despite a small sample size, there was a trend toward fewer patients with durable PFS in TCR-T-treated patients than among TIL-treated patients. Only one patient in the surviving cohort received TCR-T of the six total included in this analysis. This result could speak to the polyclonal nature of TIL targets in contrast to the monoclonal targets of TCR-T. Although never proven directly, basic tenets of cancer biology suggest that the polyclonal nature of many TIL products can limit the development of resistant tumors that lack antigen or major histocompatibility complex expression that represent a major immune evasion mechanism following treatment with a monoclonal TCR product. This phenomenon has increasing relevance with the increased availability of TCR-T, especially considering the recent FDA approval of the first TCR therapy for metastatic synovial cell sarcoma.^19^

Mechanisms of immune resistance gleaned from progressing lesions are limited in their guidance of patient selection for post-ACT interventions. However, the heterogeneity present in progressing lesions when compared with original TIL specimens, especially in the context of vetted neoantigen reactivity, is perhaps relevant to combinatorial therapies. Although HLA loss of heterozygosity and B2M loss represent common mechanisms of tumor resistance in immunotherapy, these have not been comprehensively explored after ACT TIL treatment.^13–16^ In the subset of patients explored in this cohort with WES (*n* = 4), none demonstrated clear and definitive HLA loss of heterozygosity or B2M loss, although two of the four patients did demonstrate significant decreases in the clonality of their neoantigen expression by WES. Overall, it will be important in the future to properly characterize these escape lesions to fully understand the most common mechanisms of immune escape after TIL.

A key limitation of this study is that only three patients had prior treatment with ICB before ACT, and only one had prior PD-1/PD-L1 blockade. Currently, TIL is only approved for checkpoint-refractory metastatic melanoma, and anti-PD-1 therapy represents the first line of therapy for this disease. However, of the three patients in this cohort who were ICB-refractory, two remain disease-free at last follow-up more than 5 years after their initial operation. Overall, the cohort reported herein is relatively treatment-naïve, so our results are less applicable to contemporary practice, with modern-day implementation of ACT TIL to checkpoint-refractory patients with metastatic melanoma.

Escape lesions are a well described phenomenon after ICB, and ACT can mediate responses after ICB.^7^ However, the response rate for ACT is lower in ICB-refractory patients, and the mechanisms of oligoprogression could be different in an ICB-refractory patient population.^12,20^ The lower response rate observed in anti-PD-1 refractory patients was also associated with a lower detection of neoantigen-reactive T cells in this cohort compared with anti-PD-1-naïve patients.^12^ Going forward, this will represent the bulk of patients with metastatic melanoma treated with ACT, so it must be emphasized that additional study is needed in these patients after oligoprogression. Additional limitations of this study are its small sample size, retrospective nature, and the highly selective nature of this cohort of patients. This study shows that resection of escape lesions can be an effective intervention in highly selected patients with oligoprogression after ACT but does not provide any evidence that surgery is superior or inferior to alternative salvage regimens.

In summary, we report with over 10 years of follow-up that salvage metastasectomy can provide durable disease control in highly selected patients with escape lesions after ACT. This study provides insight into the biology of melanoma progression after ACT and supports the notion that escape lesions in some patients truly represent local failures. Surgery and other local therapies should be carefully considered alongside systemic options for patients with escape lesions in a multidisciplinary setting.

## Supplementary Information

Below is the link to the electronic supplementary material.

Supplementary Material 1: **Fig. 1 **Kaplan-Meier curves showing OS and RFS for patients with oligoprogression who were resected to no evidence of disease (NED) or to non-progressing residual disease (NPRD).

Supplementary Material 2: **Fig. 2 **Representative ELISpot assay measuring interferon-gamma (IFN-y) release after co-culture of original TIL and escape lesion TIL with three different neoantigens. Two neoantigens, GATA6 and ATL2, were not found in the progressing tumor.
