# Supplementary material for: Salvage Metastasectomy After Adoptive Cell Transfer for Melanoma: Long-Term Updates
Source: Ann Surg Oncol. 2026 Mar 21;33(9):7935–43. doi: 10.1245/s10434-026-19354-1 (PMC13452880; doi:10.1245/s10434-026-19354-1)

Supplementary Figure 2

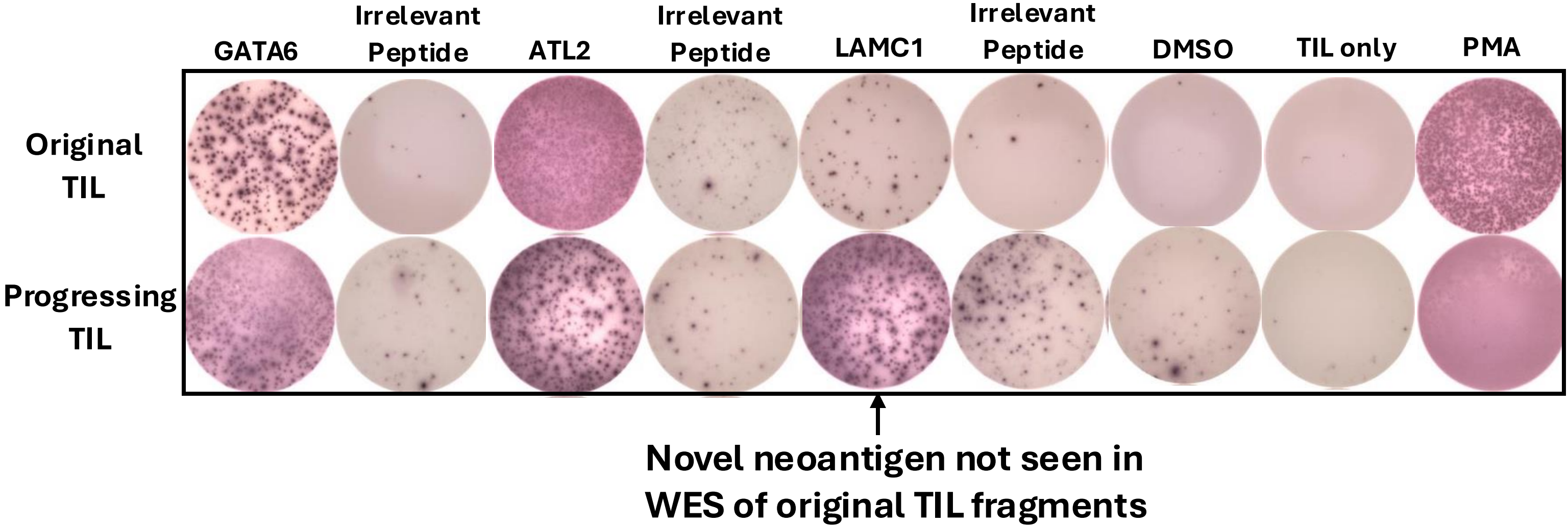

Supplement: Supplementary file 1 [file 10434_2026_19354_MOESM1_ESM.pdf]
