# Supplementary material for: Salvage Metastasectomy After Adoptive Cell Transfer for Melanoma: Long-Term Updates
Source: Ann Surg Oncol. 2026 Mar 21;33(9):7935–43. doi: 10.1245/s10434-026-19354-1 (PMC13452880; doi:10.1245/s10434-026-19354-1)
Supplement: Supplementary file 2 [file 10434_2026_19354_MOESM2_ESM.pdf]

Supplementary Figure 1

NED vs. NPRD

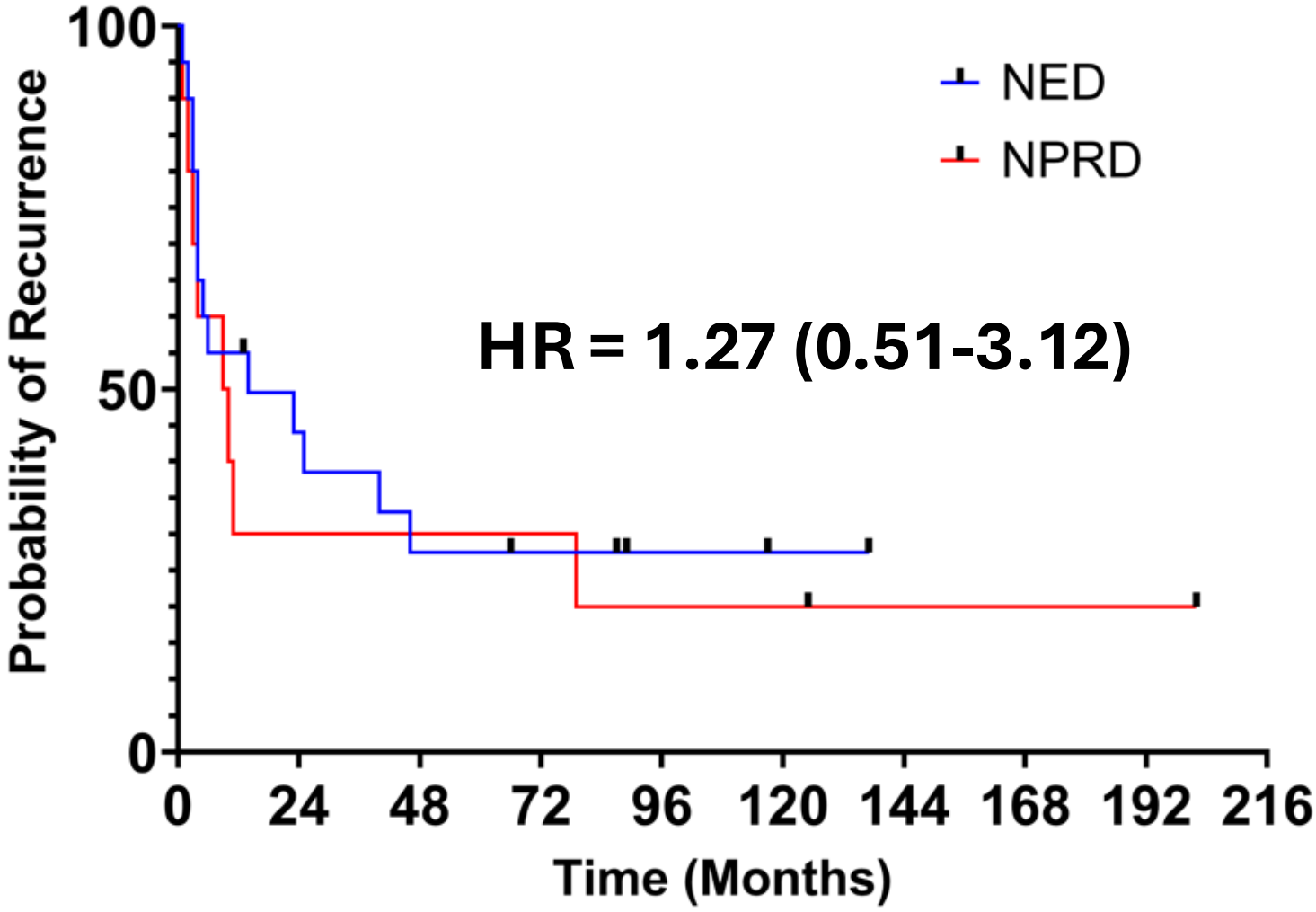

Number at Risk										
NED	20	8	5	4	2	1	0	0	0	0
NPRD	10	3	3	3	2	2	1	1	1	0

NED vs. NPRD

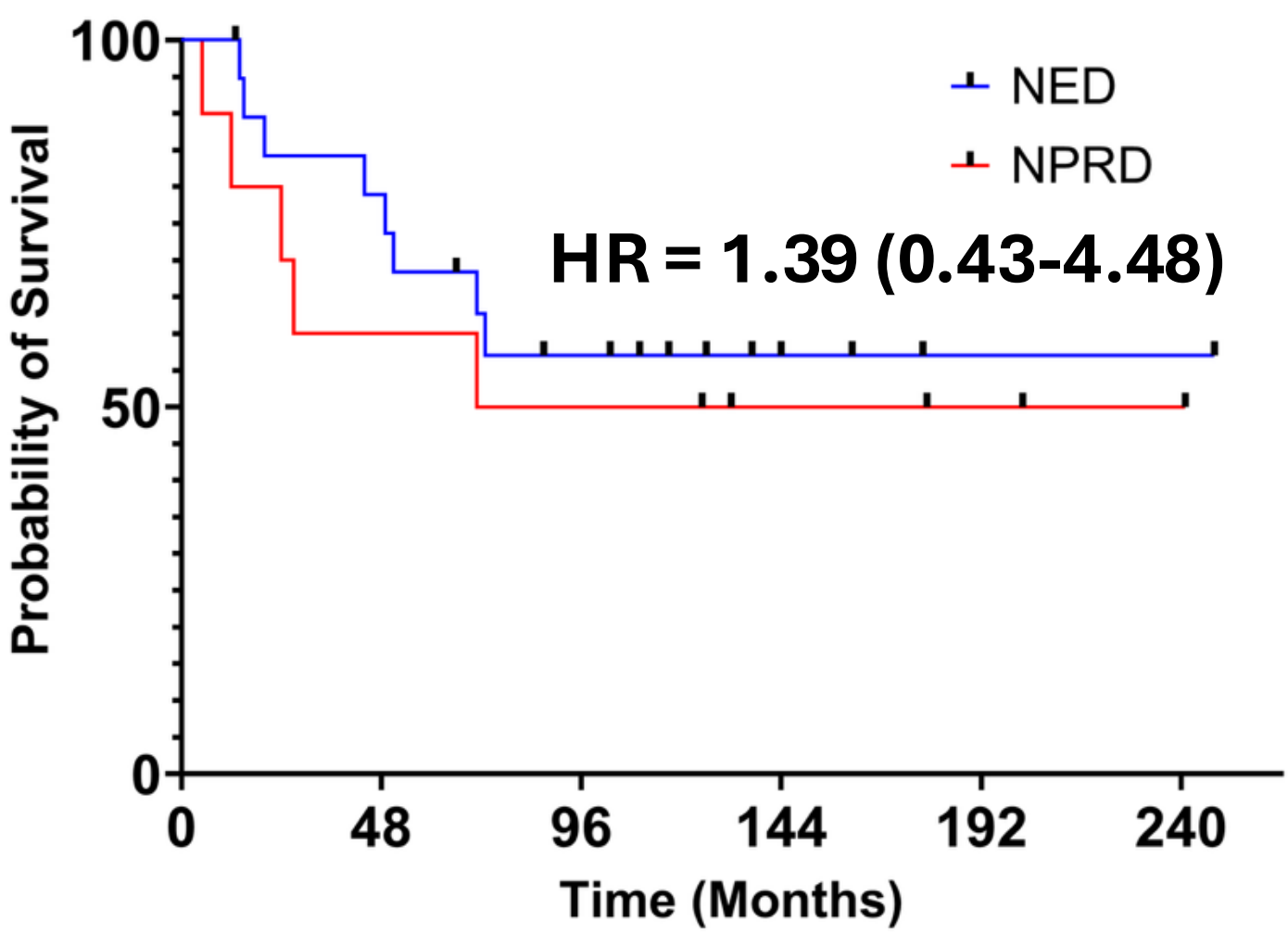

Number at Risk						
NED	20	15	9	4	1	1
NPRD	10	6	5	3	2	1
